# Progress of Research on Cognitive Impairment in Patients With Chronic Obstructive Pulmonary Disease

**DOI:** 10.62641/aep.v54i3.2207

**Published:** 2026-06-15

**Authors:** Xia Zhang, Tao Li, Hongwei Ma, Yanbing Liu

**Affiliations:** ^1^Department of Respiratory Medicine, 983rd Hospital of the Joint Logistics Support Force, 300142 Tianjin, China

**Keywords:** chronic obstructive pulmonary disease, cognitive impairment, pathogenesis

## Abstract

Cognitive impairment is a common complication in patients with chronic obstructive pulmonary disease (COPD) that substantially affects their treatment adherence, self-management ability and prognosis; it is an important extrapulmonary complication. Its pathogenesis is complex and mainly involves cerebral hypoperfusion and neuronal damage caused by chronic hypoxemia and disruption of the blood–brain barrier and neural structures by systemic inflammatory responses. Smoking, frequent acute exacerbations, coexisting cerebrovascular diseases, anxiety, depression and sleep disorders are amongst the multiple factors that contribute to and accelerate cognitive decline. This article aims to review the pathogenesis, related factors and intervention measures of cognitive impairment in patients with COPD to provide a reference for improving cognitive function in such patients.

## Introduction

Chronic obstructive pulmonary disease (COPD) is a common chronic respiratory disease characterised by persistent airflow limitation, causing a heavy disease burden worldwide [1, 2]. For a long time, clinical attention has focused on its respiratory symptoms and changes in lung function. Cognitive impairment, as an important extrapulmonary manifestation of COPD, is prevalent in patients with COPD but has not been adequately recognised [3]. A study [4] has shown that COPD is an independent risk factor for cognitive impairment. Its course is positively correlated with the risk of cognitive impairment, and the longer the course of the disease is, the higher the risk is [5]. The overall prevalence of cognitive impairment is high in patients with COPD, but the data reported by different studies vary. This variation may be related to the cognitive assessment tools used in the studies, the disease stage and severity of the included patients, lung function level, comorbidity and demographic factors (e.g., age and education level) [6]. Cognitive impairment poses a serious threat to the clinical management and prognosis of patients with COPD. Most patients rely on inhalation devices for maintenance treatment for a long time, but eventually, cognitive decline will directly affect their ability to operate the device correctly, leading to reduced treatment adherence and thus affecting disease control [7]. In addition, cognitive impairment can exacerbate the decline in patients’ motor abilities, promote a sedentary lifestyle, increase the risk of functional disability and lead to a deterioration in health status by weakening self-management abilities [8]. Given that cognitive impairment often progresses insidiously, it is easily mistaken for age-related symptoms or disease fatigue, leading to delays in diagnosis and intervention [9]. Compared with previous reviews in this field, the present work offers several novel contributions. Firstly, whilst most existing reviews described risk factors or mechanisms in isolation, we systematically integrate the core pathophysiological processes, namely, chronic hypoxia, systemic inflammation and cerebrovascular injury, and explicitly discuss their synergistic and mutually reinforcing relationships. Secondly, we critically evaluate the potential confounding effects of age, education level, cardiovascular comorbidities and commonly used medications, which have received limited attention in prior reviews. Thirdly, in the intervention section, we not only summarise available strategies but also assess the strength of evidence for each approach, identify gaps between preclinical findings and clinical application and propose an individualised, phenotype‑based framework for future research and practice. This article systematically reviews the progress of recent research in this field to provide a reference for early identification, clinical management and future research.

## Materials and Methods

### Literature Search Strategy

This review was conducted in accordance with the Preferred Reporting Items for Systematic Reviews and Meta-Analyses (PRISMA) guidelines. We systematically searched PubMed, Google Scholar and Web of Science electronic databases from January 2021 to May 2026. The search strategy employed Boolean operators to combine medical subject headings (MeSH) terms with free-text words. The complete search syntax for each database is provided below to ensure transparency and reproducibility.

#### PubMed

“Pulmonary disease, chronic obstructive” (MeSH) OR “chronic obstructive pulmonary disease” OR “COPD” AND “cognitive impairment” (MeSH) OR “cognitive dysfunction” OR “cognitive decline” OR “dementia” OR “memory impairment” OR “executive function” OR “attention” AND “pathogenesis” OR “etiology” OR “risk factor” OR “inflammation” OR “hypoxia” OR “cerebrovascular” OR “intervention” OR “treatment” OR “management” OR “therapy” OR “rehabilitation”. Filters: publication date from 2021 to 2026. English, humans.

#### Web of Science

TS = “chronic obstructive pulmonary disease” OR “COPD” AND “cognitive impairment” OR “cognitive dysfunction” OR “cognitive decline” OR “dementia” OR “memory impairment” OR “executive function” OR “attention” AND “pathogenesis” OR “etiology” OR “risk factor” OR “inflammation” OR “hypoxia” OR “cerebrovascular” OR “intervention” OR “treatment” OR “management” OR “therapy” OR “rehabilitation”. Timespan: 2021–2026. Indexes: SCI-EXPANDED, SSCI, ESCI. Language: English.

#### Google Scholar

“Chronic obstructive pulmonary disease” OR “COPD” AND “cognitive impairment” OR “cognitive decline” OR “dementia” AND “pathogenesis” OR “risk factors” OR “intervention” OR “treatment”. Custom range: 2021–2026. Results sorted by relevance, first 200 records screened.

### Inclusion and Exclusion Criteria

Inclusion criteria: (1) the study subjects were patients with a confirmed diagnosis of COPD (aged ≥40 years); (2) the research topics involved the epidemiology, risk factors, pathogenesis, assessment methods, prevention, or interventions for cognitive impairment; (3) the study types included randomised controlled trials, cohort studies, case-control studies, cross-sectional studies, systematic reviews and meta-analyses; and (4) the studies used Chinese and English languages. Exclusion criteria: (1) non-Chinese or non-English language publications; (2) case reports, conference abstracts, commentaries, editorials and letters; (3) publications of low research quality or with incomplete data; and (4) duplicate publications.

### Literature Screening Process

**Fig. 1.**
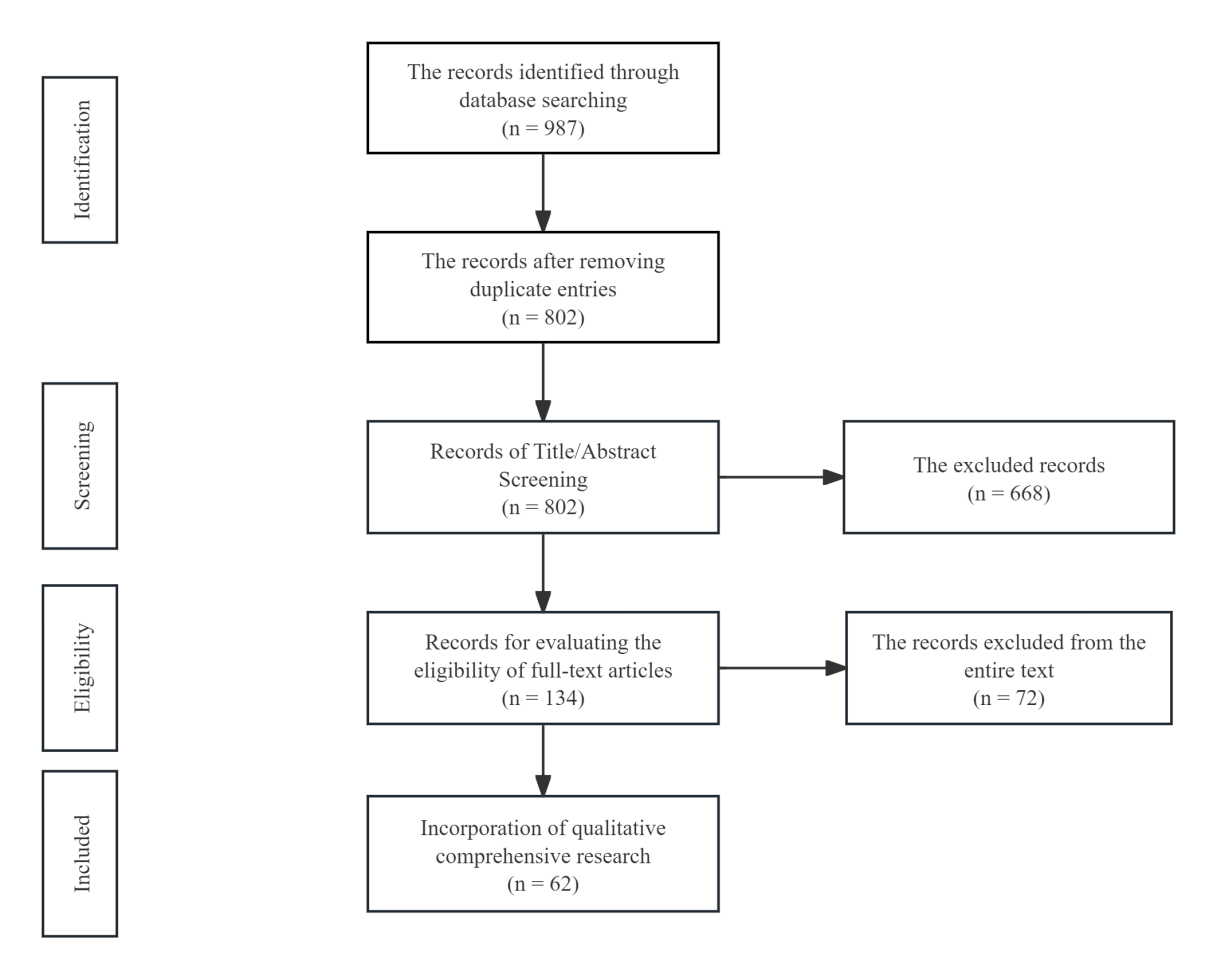
**PRISMA flowchart**.

Two researchers independently conducted literature screening and data extraction. Disagreements were resolved through discussion or adjudication by a third researcher. The screening process involved an initial review of titles and abstracts to exclude clearly irrelevant studies, followed by full-text reading for secondary screening. The data extracted included authors, publication year, study type, sample size, patient characteristics, cognitive assessment tools, key findings and conclusions. The literature screening process is shown in Fig. 1 (PRISMA flowchart). A total of 62 articles were included for the review.

## Mechanism of Occurrence and Related Factors

### Core Pathophysiological Mechanisms

#### Chronic Hypoxia and Inadequate Cerebral Perfusion

Chronic hypoxemia is one of the most common pathophysiological states in patients with COPD, and its effects on the brain are direct and multifaceted [10]. With regard to direct neurotoxicity, given that the brain is an organ that is highly dependent on an adequate supply of oxygen, neurons are extremely sensitive to oxygen deprivation [11]. Long-term oxygen deficiency leads to mitochondrial dysfunction and reduced adenosine triphosphate (ATP) production, triggering oxidative stress and excitatory amino acid toxicity and ultimately leading to neuronal apoptosis and loss, especially affecting brain regions closely related to learning and memory, such as the hippocampus [12]. With regard to cerebral hemodynamic disturbances, cerebral blood vessels compensatorily dilate at the early stages of hypoxia to maintain brain oxygen supply. However, in the chronic course of COPD, this compensatory mechanism may be exhausted or disordered, leading to chronic cerebral inadequacy [13]. In addition, hypoxia can induce endothelial dysfunction and hypercoagulable state, further damaging microcirculation [14]. Neuroimaging studies have further revealed that stable patients with COPD patients exhibit substantial grey matter density reduction in cognitively related brain regions, such as the limbic system, right rectus, left precentral gyrus, bilateral superior temporal gyrus and anterior insula. These structural changes are correlated with the degree of decrease in blood oxygen partial pressure; the more substantial grey matter atrophy is, the worse the patient’s visuospatial memory and executive function tend to be [15]. Moreover, patients with COPD generally have reduced cerebral perfusion, and the severity of hypoxemia is positively correlated with cognitive impairment. Patients with blood oxygen saturation below 88% have a considerably increased risk of cognitive impairment, especially in cognitive domains, such as language memory and delayed recall, suggesting that insufficient cerebral perfusion can cause cerebral ischemia and subcortical atrophy and that the degree of reduction is directly related to the severity of cognitive deficits [16, 17].

#### Systemic Inflammation and Neurovascular Unit Damage

COPD is a systemic inflammatory disease. Persistent chronic inflammation in the lungs produces many pro-inflammatory mediators, such as interleukin-6 (IL-6), IL-8, tumour necrosis factor-α (TNF-α) and C-reactive protein (CRP). These factors enter the systemic circulation, break through the fragile blood–brain barrier, or indirectly affect the central nervous system by activating peripheral immune cells [18, 19]. The mechanisms by which they damage the brain include (a) disruption of the blood–brain barrier: inflammatory factors can upregulate the expression of matrix metalloproteinases, degrade basement membrane components, increase the permeability of the blood–brain barrier and facilitate the entry of neurotoxic substances into the brain; (b) activation of microglia: after the central immune cells (microglia) are activated, they transform into a pro-inflammatory phenotype, release a large number of neurotoxic substances and form a vicious cycle of neuroinflammatory environment [20]; and (c) damage to neurovascular coupling: the neurovascular unit is the functional unit for the regulation of neuronal activity and local blood flow, and inflammation can damage the function of endothelial cells, pericytes and astrocytes in this unit, resulting in the inability of blood flow to increase accordingly when neuronal activity increases, thereby causing functional hypoxia [21]. The aforementioned inflammatory process directly promotes the occurrence and development of cerebrovascular lesions. CRP, as one of the key mediators in this process, is closely associated with cognitive decline when its level increases and can directly inhibit endothelial function and promote atherosclerosis [22]. A study [23] has confirmed that COPD-related chronic inflammation and hypoxia synergistically accelerate atherosclerosis and specifically damage the structure and function of small cerebral vessels. Peng *et al*. [24] found through functional magnetic resonance imaging that even in patients with COPD in the stable phase, substantial neurovascular changes have already occurred in their brains, mainly manifested as abnormal cerebral blood flow in key brain regions and impaired neurovascular coupling function, suggesting that chronic inflammation and hypoxia could damage the regulatory function of cerebral blood vessels and the health of neurovascular units before the appearance of obvious clinical symptoms. Furthermore, COPD has been confirmed as an independent risk factor for cerebral microbleeds, further indicating that its pathological process can promote widespread microvascular lesions [25].

#### Exacerbating Effect of Smoking on the Abovementioned Mechanisms

As the primary pathogenic factor for COPD, smoking“s effect extends beyond the initial stages of the disease, permeating the entire process of cognitive impairment and exerting a synergistic, additive effect with the mechanisms described above [26]. Tobacco smoke contains various neurotoxic compounds, including nicotine, carbon monoxide, lead and mercury. These compounds can rapidly cross the blood–brain barrier [27]. Smoking exacerbates existing lung disease, leading to a severe decline in lung function and hypoxemia. Moreover, long-term smoking considerably increases the concentration of carbon monoxide in the brain. Given that carbon monoxide has a much higher affinity for hemoglobin than oxygen, it easily forms carboxyhemoglobin, leading to a decrease in the blood’s oxygen-carrying capacity and causing chronic hypoxia in brain tissue [28, 29]. Nicotine, as the main active ingredient in tobacco, acts extensively on nicotine acetylcholine receptors distributed throughout the brain, affecting various neurotransmitter systems, such as norepinephrine and dopamine, thereby interfering with cognitive processes, including attention, memory and executive function [30]. Although short-term nicotine exposure may exert some cognitive-activating effects, long-term smoking disrupts the cognitive control network dependent on the prefrontal–striatal–thalamic circuit, increasing the risk of dementia [31]. A neuroimaging study [32] has further confirmed that comp ared with non-smokers, long-term smokers generally have smaller grey matter volume and lower density, indicating that white matter structure is also affected. Although some brain regions may experience a temporary increase in volume because of vasogenic edema, the total volume of white matter in the whole brain tends to decrease. These structural changes are closely related to cognitive decline, and their reversibility is currently unclear.

### Clinical and Behavioural Factors

#### Acute Exacerbation of COPD

COPD exacerbations are often triggered by factors, such as infection, leading to a rapid deterioration of respiratory symptoms and requiring adjustments to the original treatment plan. During this period, the patient’s systemic inflammatory response is substantially enhanced compared with that in the stable phase, exerting a notable, persistent negative effect on cognitive function [33]. A study [33] has shown that the cognitive function of patients during acute exacerbations is much lower than that during the stable phase, and this impairment shows limited improvement even three months after the condition stabilises, suggesting that its effects may be persistent. Research [34] has indicated that cognitive function exhibits specific changes with disease stages. From the acute exacerbation phase to the remission phase, visual construction, attention, language, abstract thinking, delayed recall and orientation may fluctuate; after transitioning from acute exacerbation to a stable phase, patients have increased potential for recovery in cognitive functions in certain areas, such as naming, attention, language, abstract thinking and delayed recall. Individual acute exacerbations impair cognitive function, and the cumulative number of repeated exacerbations is closely correlated with cognitive decline; the higher the frequency and severity of acute exacerbations are, the greater the risk of cognitive impairment progression is [35]. A study [36] has shown that patients with COPD in the acute exacerbation phase have poor cognitive function, and magnetic resonance imaging scans indicated that patients with COPD in the acute exacerbation phase have damage to their brain white matter microstructure, manifested as ventricular enlargement and increased volume of white matter lesions.

#### Comorbid Affective and Sleep Disorders

Anxiety and depression are not only common comorbidities of COPD but also independent risk factors for cognitive impairment [37]. The respiratory distress, functional decline and recurrent acute exacerbations caused by the disease impose continuous psychological stress on patients, triggering or exacerbating affective disorders. Symptoms of depression and anxiety can directly affect attention, executive function and memory and may further exacerbate cognitive decline through pathways, such as activation of the hypothalamic–pituitary–adrenal axis, increased inflammation levels and social withdrawal [38]. A study [39] has shown that patients with COPD and affective disorders experience rapid cognitive decline, and their risk of developing mild cognitive impairment or even dementia is substantially increased. Sleep disorders are prevalent in COPD patients and manifest as insomnia, sleep fragmentation, hypoxemia and frequent awakenings. More than half of patients experience poor sleep quality, and a large proportion of them also have obstructive sleep apnea (OSA) [40]. Sleep disorders can impair cognition in many ways. Firstly, fragmented sleep and reduced deep sleep directly impair memory consolidation and the clearance of metabolic waste from the brain. Secondly, intermittent hypoxia caused by OSA can aggravate neuronal damage and oxidative stress. Thirdly, long-term sleep deprivation can promote the deposition of β-amyloid protein and accelerate neurodegeneration [41].

#### Other Confounding Factors

Age and Education Level

Age is a risk factor for neurodegeneration and vascular aging. COPD and aging may have a synergistic accelerating effect on hypoxia, inflammation and other areas [42]. Higher education levels may promote neuroplasticity, enhance the connectivity and efficiency of brain networks and thus effectively mobilise alternative neural networks and cognitive resources in response to COPD-related brain injury, delaying the manifestation of cognitive impairment at the behavioural level [5]. Therefore, even with similar levels of brain pathology, patients with higher education levels who have COPD may maintain good cognitive function for a long period, leading to delayed recognition of their clinical cognitive impairment [6].

Cardiovascular Comorbidities

Hypertension, diabetes, atrial fibrillation and heart failure are common comorbidities of COPD and clear risk factors for vascular cognitive impairment [43]. They can directly lead to cerebral small vessel disease, microinfarction and amyloid angiopathy. COPD-related systemic inflammation and hypoxia may accelerate the progression of these cardiovascular diseases, indirectly increasing the risk of cognitive impairment [25].

Drug Treatment

Drugs commonly used for COPD and its comorbidities, such as anticholinergic drugs, benzodiazepines, certain antihistamines and long-term high-dose systemic glucocorticoids, have side effects that can cause delirium or impair attention and memory function [44].

## Intervention Measures

### Drug Treatment

#### Roflumilast

Roflumilast, a selective phosphodiesterase-4 inhibitor, is primarily used clinically to reduce the risk of acute exacerbations in COPD. Bhat *et al*. [45] demonstrated through animal studies that roflumilast improves learning and memory abilities in mice with sleep-deprivation-induced cognitive impairment. Potential mechanisms involve suppressing neuroinflammation, modulating cyclic adenosine monophosphate signalling pathways and promoting brain-derived neurotrophic factor expression. Furthermore, roflumilast has demonstrated cognitive enhancement effects on healthy adults, healthy elderly individuals, patients with mild cognitive impairment and patients with schizophrenia [46]. The aforementioned findings primarily originate from animal models or non-COPD populations. Questions regarding roflumilast’s optimal intervention timing, suitable patient populations and long-term safety remain to be further investigated.

#### Statins

Aside from having lipid-lowering effects, statins (e.g., simvastatin and atorvastatin) perform multifaceted actions and have anti-inflammatory, antioxidant and endothelial function-improving properties. Theoretically, they may exert their effects by attenuating the systemic inflammation associated with COPD [47]. Research has shown that acute COPD exacerbations correlate with cognitive impairment potentially through amplified systemic inflammation [25]. Concurrently, a clinical study has suggested that statins exert anti-inflammatory effects by reducing inflammatory markers, such as CRP and IL-6 [48]. Presently, evidence on the cognitive benefits of statins in patients with COPD is primarily derived from observational studies and is insufficient to support the use of statins as an independent indication for cognitive impairment.

#### Pharmacological Management of Comorbid Dementia

For patients with COPD and Alzheimer’s disease or other dementias, cholinesterase inhibitors (donepezil, rivastigmine and galantamine) and memantine are commonly prescribed. Theoretically, cholinesterase inhibitors may exacerbate airway obstruction by increasing cholinergic activity. However, population-based cohort studies have shown that newly initiating cholinesterase inhibitors in patients with COPD and dementia does not substantially increase the risk of COPD-related emergency department visits or hospitalisations, suggesting respiratory safety within clinically acceptable limits [49]. As an N-methyl-D-aspartate receptor antagonist, memantine exerts neuroprotective effects by preventing glutamate excitotoxicity. Its use is highly prevalent in patients with Alzheimer’s disease and with coexisting asthma/COPD to avoid potential antagonism with inhaled anticholinergic medications [50]. In clinical practice, individualised selection should be based on the patient’s dementia subtype, disease stage, COPD severity and concomitant medications.

#### DL-3-n-Butylphthalide

Animal studies have indicated DL-3-n-butylphthalide promotes hippocampal neurogenesis and inhibits abnormal neural network remodelling, suggesting potential cognitive protective effects under pathological conditions, such as chronic hypoxia [51].

### Non-Pharmacological Intervention Methods

#### Oxygen Therapy

Long-term oxygen therapy is the fundamental treatment for correcting chronic hypoxaemia in patients with COPD. According to current guidelines, the indications for long-term oxygen therapy are as follows: arterial partial pressure of oxygen (PaO_2_) ≤ 55 mmHg at rest or PaO_2_ 56–59 mmHg accompanied with pulmonary hypertension, peripheral oedema, or polycythaemia. The standard recommended flow rate is 1–2 L/min, which is adjusted to achieve peripheral oxygen saturation (SpO_2_) ≥ 90% at rest, with a daily oxygen therapy duration of not less than 15 hours [52, 53]. Annaka’s study [54] revealed that patients with COPD who have adhered to standardised long-term oxygen therapy have a slow rate of cognitive decline and good overall neurological prognosis, suggesting that long-term continuous oxygen therapy may reduce the neurological damage and cognitive decline caused by hypoxia by correcting hypoxemia and improving oxygen supply to brain tissue. Thakur *et al*. [55] demonstrated that amongst individuals with COPD, those using home oxygen have much lower odds of cognitive impairment (OR = 0.14, 95% CI: 0.07–0.27) after adjusting for potential confounders, suggesting that long-term oxygen therapy may confer neuroprotective effects. However, in clinical practice, poor adherence to long-term oxygen therapy persists because of economic burdens, device adaptability and psychosocial factors. Approximately 30%–65% of patients fail to achieve the recommended 15-hour daily oxygen duration, considerably compromising therapeutic efficacy [56]. Therefore, clarifying the indications for oxygen therapy and improving patients’ treatment adherence are key links in achieving cognitive protection.

#### Pulmonary Rehabilitation

Pulmonary rehabilitation is a comprehensive, multidisciplinary intervention program that includes personalised exercise training, health education and psychological support. A study [57] has shown that short-term intensive training and structured courses lasting several weeks can substantially improve patients’ overall cognitive level, especially in terms of language fluency, visuospatial ability and executive function. In addition, even patients with COPD and mild cognitive impairment can benefit from pulmonary rehabilitation. Study [58] has also found that patients discharged after acute exacerbations who do not receive pulmonary rehabilitation have difficulty recovering their cognitive function naturally, highlighting the importance of early rehabilitation intervention. Although long-term exercise helps maintain cognitive level, ensuring sustained participation is a challenge to the achievement of long-term benefits.

#### Cognitive Training

Cognitive training aims to improve specific cognitive domains, such as memory, attention and executive function, through targeted exercises. Its effectiveness in patients with COPD remains inconsistent. A study [59] has shown that short-term, structured cognitive training can improve patients’ cognitive test scores; however, other studies [53] have reported that even several months of intensive working memory training fail to substantially improve patients’ overall cognitive function. These differences in effectiveness may be related to the design, intensity and duration of the training program and patient differences. Compared with pulmonary rehabilitation, cognitive training as a standalone intervention is weaker, and its universal efficacy remains unclear. Future research may need to explore whether combined interventions integrating cognitive training with physical exercise yield synergistic effects [60].

#### Nutritional Intervention

Specific dietary patterns and nutritional supplements are potential intervention strategies. Existing evidence suggests that healthy dietary patterns, such as Mediterranean and Dietary Approaches to Stop Hypertension (DASH) diets, help delay cognitive decline in the general elderly population [61]. In addition, specific combinations of nutrients, such as n-3 polyunsaturated fatty acids, uridine and B vitamins, have been shown to exert a positive effect on cognitive function [62]. Such nutritional interventions are easy to integrate into daily life and highly feasible. However, caution is warranted regarding their applicability in the COPD population. The aforementioned evidence is primarily derived from studies involving the general elderly population or patients with mild cognitive impairment. High-quality research remains lacking to validate the efficacy, appropriate protocols and specific mechanisms of action of these interventions within the specific COPD cohort. Patients with COPD frequently present with special conditions, such as malnutrition and sarcopenia; nutritional interventions must therefore be individually tailored in consideration of their overall systemic condition.

## Conclusions

With the increasing aging of the society, the prevalence of COPD is rising, and its common comorbid cognitive impairment is a key issue affecting patient prognosis. Clinical practice should emphasise early screening and intervention of cognitive function in patients with COPD to mitigate the disease’s adverse effects on self-management and overall prognosis. Currently, no consensus has been reached regarding intervention strategies for COPD-related cognitive impairment. Pulmonary rehabilitation is the core intervention with the strongest evidence base, and oxygen therapy requires focus on optimising protocols and ensuring adherence. Pharmacological treatment, cognitive training and nutritional interventions remain in the exploratory phase. Future research should focus on defining the indications, timing and dosage for each intervention; exploring synergistic effects through combined therapeutic approaches validated by multicentre randomised controlled trials and long-term follow-up; and integrating objective assessments, such as neuroimaging, to elucidate the structural and functional underpinnings of cognitive impairment, thereby providing sufficient robust evidence for clinical practice.

## Availability of Data and Materials

The datasets used and/or analysed during the current study were available from the corresponding author on reasonable request.
